# Development and validation of the prognostic model based on autophagy-associated genes in idiopathic pulmonary fibrosis

**DOI:** 10.3389/fimmu.2022.1049361

**Published:** 2022-12-12

**Authors:** Guoqing Fan, Jingjing Liu, Zhen Wu, Caiyu Li, Ying Zhang

**Affiliations:** ^1^ Department of Respiratory Medicine and Critical Care, Beijing Hospital, National Center of Gerontology, Institute of Geriatric Medicine, Chinese Academy of Medical Sciences, Beijing, China; ^2^ Graduate School of Peking Union Medical College, Beijing, China; ^3^ Department of Respiratory and Critical Care Medicine, Shandong Provincial Hospital Affiliated to Shandong First Medical University, Jinan, Shandong, China; ^4^ Department of Respiratory & Critical Care Medicine, The Second Hospital, Cheeloo College of Medicine, Shandong University, Jinan, Shandong, China

**Keywords:** IPF, autophagy, prognosis, bioinformatics, immune infiltration

## Abstract

**Background:**

Idiopathic pulmonary fibrosis (IPF) is a chronic progressive interstitial lung disease. Many studies suggest that autophagy may be related to disease progression and prognosis in IPF. However, the mechanisms involved have not been fully elucidated.

**Methods:**

We incorporated 232 autophagy-associated genes (AAGs) and two datasets, GSE28042 and GSE27957, from the GEO database. Univariate Cox analysis and least absolute shrinkage and selection operator (LASSO) regression were used to construct the autophagy-associated prognostic model. Gene Ontology (GO) and Kyoto Encyclopedia of Genes and Genomes (KEGG) analyses were performed to investigate the functions of these autophagy-associated genes. CIBERSORT algorithm was used to calculate the immune cell infiltration between patients in the high-risk score and low-risk score groups. Quantitative Real-Time Polymerase Chain Reaction (qRT-PCR) was performed to explore the mRNA expression of five genes in the autophagy-associated risk model.

**Results:**

We constructed a 5-autophagy-associated genes signature based on Univariate Cox analysis and LASSO regression. In our autophagy-associated risk model, IPF patients in the high-risk group demonstrated a poor overall survival rate compared to patients in the low-risk group. For 1-, 2-, and 3-year survival rates, the AUC predictive value of the AAG signature was 0.670, 0.787, and 0.864, respectively. These results were validated in the GSE27957 cohort, confirming the good prognostic effect of our model. GO and KEGG pathway analyses enriched immune-related pathways between the high-risk and low-risk groups. And there was also a significant difference in immune cell infiltration between two groups. And the results of qRT-PCR showed that the expression levels of *FOXO1*, *IRGM*, *MYC*, and *PRKCQ* were significantly decreased in the Peripheral Blood Mononuclear Cell (PBMC) of IPF patient samples.

**Conclusion:**

Our study constructed and validated an autophagy-associated risk model based on *MYC*, *MAPK1*, *IRGM*, *PRKCQ*, and *FOXO1*. And those five genes may influence the progression of IPF by regulating immune responses and immune cells.

## Introduction

Idiopathic pulmonary fibrosis (IPF) is a chronic progressive interstitial lung disease (1). IPF occurs most often in middle-aged and elderly people and is associated with progressive cough and dyspnea and reduced quality of life (2). IPF patients have a very poor prognosis, with a median survival of approximately 2-3 years after diagnosis (3). Despite the growing research on IPF, the cause of IPF is unknown and the pathogenesis involves multiple factors, including aging, environmental exposure, smoking, and chronic viral infections (1, 4, 5). Lung transplantation is currently the only treatment for IPF that can reverse the disease process of IPF. However, the limited source of donors, the high cost of the procedure, and the high number of IPF patients with comorbid underlying diseases are factors that limit the use of lung transplantation in the treatment of IPF patients (6). Pirfenidone and nintedanib have been recommended by guidelines for the treatment of IPF (6). Although pirfenidone and nintedanib delayed the decline in lung function and improved progression-free survival in IPF patients, they did not significantly improve overall mortality and acute exacerbations in IPF patients (7–9). Therefore, it is important to explore new molecular mechanisms of IPF pathogenesis and to construct new predictive models related to IPF prognosis.

Autophagy is a cellular degradation and recycling process that is highly conserved in eukaryotes (10). In mammalian cells, there are three major types of autophagy: microautophagy, macroautophagy, and chaperone-mediated autophagy (CMA). Although each is morphologically distinct, all three ultimately transport cargo to lysosomes for degradation and recycling (11). Autophagy plays an important role in cell survival and maintenance by degrading organelles, proteins, and macromolecules in the cytoplasm, as well as recycling degradation products. However, uncontrolled autophagy can lead to apoptosis. Therefore, dysfunction of this process can lead to the development of a variety of diseases (12, 13). Autophagy also exhibits an important role in IPF. Autophagy can slow down the pathological progression of IPF by regulating apoptosis of fibroblasts and senescence of alveolar epithelial cells (14, 15). In lung epithelial cells and lung fibroblasts from IPF patients, autophagy-associated pathways (including macroautophagy and mitophagy) were attenuated in both IPF mouse models and fibroblasts activated with TGFβ1 (16).IL-37 can reduce the progression of IPF by inhibiting TGF-β1 signaling and enhancing IPF fibroblast autophagy to attenuate lung fibrosis in mice (17). However, excessive autophagy of macrophages can promote the proliferation and migration of fibroblasts and exacerbate the progression of silicotic fibrosis (18). Autophagy is involved in the generation of inflammation and fibrosis formation in lung tissue by regulating neutrophil extracellular traps (19). All these studies suggest that autophagy may be related to disease progression and prognosis in IPF. However, strong evidence for the correlation between autophagy and IPF prognosis is still lacking. Therefore, it is crucial to construct a prognostic model of IPF with autophagy-associated genes.

In this study, we constructed an autophagy-associated genetic prognostic model for IPF using univariate Cox analysis and least absolute shrinkage and selection operator (LASSO). Subsequently, we validated the model using a cohort of IPF patients from the GEO database and a cohort of collected IPF patients. Finally, we explored the relationship between the autophagy risk model and inflammatory cells. Our study contributes to the understanding of the link between autophagy and IPF prognosis. Autophagy-associated genes may be potential therapeutic targets for IPF patients.

## Methods

### Data acquisition and processing

We downloaded 232 autophagy-associated genes (AAGs) from the Human Autophagy Database website (http://www.autophagy.lu/index.html). We downloaded two datasets, GSE28042 and GSE27957, from the GEO database. The GSE28042 dataset contains PBMC of 75 IPF patients and 19 normal controls. The GSE27957 dataset contains PBMC of 45 IPF patients. The basic information of the patients in GSE28042 and GSE27957 is shown in **Table 1**. The GSE28042 dataset is used as the training set and the GSE27957 dataset is used as the validation set. The principal component analysis (PCA) is used to evaluate the repeatability of the GSE28042 dataset. We used the “limma” package by R to identify differentially expressed genes (DEGs) between the IPF patient group and the control group according to p-values< 0.05 and absolute log2-fold changes > 0.2 in the GSE28042 dataset.

**Table 1:** Basic information of the patients in GSE28042 and GSE27957.

	GSE28042	GSE28042	GSE27957
	IPF (N=75)	Control (N=19)	IPF (N=45)
Age	69.0 ± 8.1	53.3 ± 10.0	67.1 ± 8.1
Sex	N=75	N=19	N=45
Male	52 (69.3%)	12 (63.2%)	40 (88.9%)
Female	23 (30.7%)	7 (36.8%)	5 (11.1%)

### PPI and correlation analyses of autophagy-associated DEGs

The STRING database (https://string-db.org/) and Cytoscape software (version 3.9.0) were used to analyze the protein-protein interaction (PPI) among autophagy-associated DEGs.

### Autophagy-Associated prognostic model construction

First, we selected genes in which DEGs and AAGs overlapped. Subsequently, univariate Cox analysis was performed on the duplicated AAGs to select genes associated with prognosis. To further narrow the AAGs scope, we performed the least absolute shrinkage and selection operator (LASSO) regression algorithm in the training set, 10-fold cross-validation was used, and the “glmnet” package by R was used for the analysis. The risk score publicized to predict the prognosis of IPF patients was calculated as follows:


$$Riskscore=\sum_{i=0}^{N}\left(\beta i\times Expi\right)$$

where *N* is the number of selected prognostic AAGs, *βi* is the regression coefficient derived from the LASSO regression model coefficients, and *Expi* represented the gene expression level for the identified AAGs. We divided IPF cases into high-risk score group and low-risk score group based on the median risk score. Kaplan-Meier survival analysis was used to assess the relationship between overall survival and the AAGs correlation model. We used the time-dependent receiver operating characteristic (ROC) curves and the corresponding areas under the curve (AUC) values for assessing the prognostic value of the AAGs-related risk model. The prognostic model was evaluated using the “survminer” and “timeROC” packages by R.

To predict the prognosis of IPF patients, we used the training set to create a nomogram predicting 2-year and 3-year survival rates for IPF patients using the “rms” package by R. Subsequently, we used the calibration curve to evaluate the predictive efficiency of the nomogram.

### Functional enrichment analysis

To explore the molecular mechanisms and pathways that are different between patients with high and low-risk scores, we performed Gene Ontology (GO) and Kyoto Encyclopedia of Genes and Genomes (KEGG) analysis using the “clusterProfiler” package by R.

### Immune infiltration analysis

We used the CIBERSORT algorithm to calculate the immune cell infiltration between patients in the high-risk score and low-risk score groups. CIBERSORT can calculate the composition and score of 22 immune cell subpopulations in clinical samples by gene expression profiling (20).

### Validation of the prognostic model

We used data of 45 IPF patients from GSE27957 to validate the accuracy of the AAGs-related risk model’s predictive ability for IPF prognosis. We also used qRT-PCR to validate the expression levels of five genes in our prognostic model. We collected PBMC samples from IPF patients attending the Second Hospital of Shandong University from January 2022 to June 2022. All IPF patients were diagnosed by high-resolution computed tomography (HRCT) and met the official ATS/ERS/JRS/ALAT clinical practice guideline for 2022 (21). Eleven IPF patients and six healthy individuals of similar age were included in our validation cohort.

### PBMC obtain, RNA extraction and quantitative real-time polymerase chain reaction

First, we added patient or control blood on the top of the lymphocyte separation solution slowly. After centrifugation at 700g for 30 min, the PBMC layer in the middle of the liquid was carefully aspirated. After resuspension of PBMC using PBS, we added Red Blood Cell (RBC) lysis buffer and leave at room temperature for 5 min. And we obtain the PBMC after the centrifugation at 300g for 5min.We extracted total RNA from PBMC using TRIzol (Invitrogen). subsequently, we performed cDNA reverse transcription and qRT-PCR using RT reagent Kit (Takara). The primer sequence is summarized in **Supplementary Data**.

### MRC-5 cell culture and western blotting

The Human lung fibroblast MRC-5 cells were obtained from Peking Union Medical College. MRC-5 cells were maintained in Minimum Essential Medium with 10% serum, and 1% penicillin and streptomycin. Cells were prepared for the next western blotting analysis after incubation with 10 ng/ml TGF-β for 48 hours. The protein extraction and western blotting experiments were carried out using standard protocols.

### Statistical analysis

We used R software (R x64 version 4.1.2) and GraphPad Prism 9 to analyze all the data. Statistical analysis for multiple group data was conducted using the Analysis of Variance (ANOVA) method followed by a Student t-test or Wilcoxon rank-sum test for the comparison between two groups. We considered the p-value<0.05 as statistically significant.

## Results

### Differential expression of autophagy-associated genes

**Figure 1:**
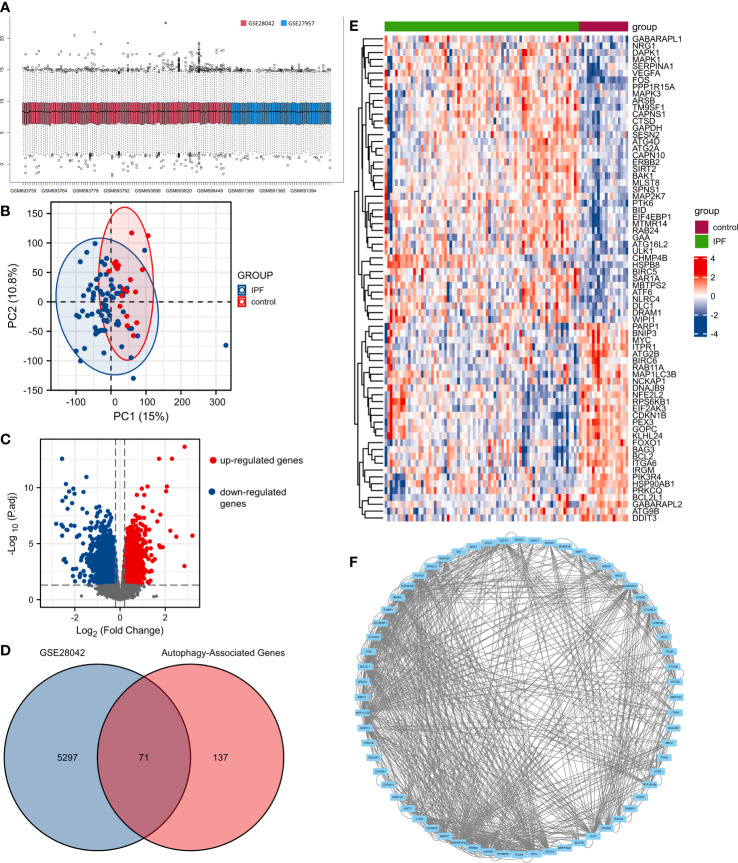
The differentially expressed autophagy-associated genes (AAGs) in idiopathic pulmonary fibrosis. **(A)** Data normalization of GSE28042 and GSE27957. **(B)** The principal component analysis (PCA) of GSE28042 dataset. **(C)** Volcano plot of differentially expressed genes in the cohort of GSE28042. **(D)** Intersection of 5368 differentially expressed genes with 208 autophagy-associated genes. **(E)** Heatmap of 71 autophagy-associated genes in IPF patients and controls. **(F)** The PPI network of autophagy-associated DEGs.

GSE28042 and GSE27957 datasets were normalized (**Figure 1A**). The result of PCA showed that GSE28042 had a good data repeatability (**Figure 1B**). We identified 5368 differentially expressed genes in the cohort of GSE28042 in the GEO database, of which 2342 genes were up-regulated and 3026 genes were down-regulated (**Figure 1C**). Based on 232 autophagy-associated genes, we identified 208 autophagy-associated genes in the expression array of GSE28042. We obtained 71 genes by taking the intersection of 5368 differentially expressed genes with 208 autophagy-associated genes (**Figure 1D**). The expression of 71 autophagy-associated genes in IPF patients and controls is displayed in the heatmap (**Figure 1E**). The PPI analysis demonstrated the interrelationship between autophagy-associated DEGs. The PPI network contained 64 nodes and 367 edges (**Figure 1F**).

### Identification of prognostic risk model of autophagy-associated genes in IPF

**Figure 2:**
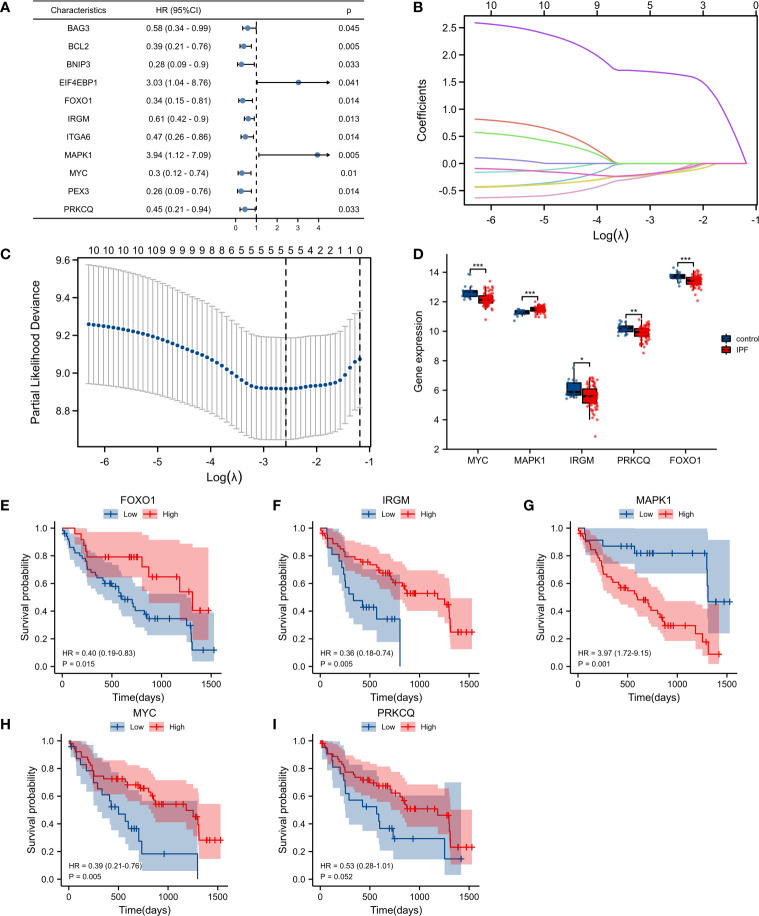
Construction of the autophagy-associated prognostic model. **(A)** Univariate Cox analysis identified genes related to the prognosis of IPF patients. **(B, C)** Screening of characteristic genes by LASSO regression analysis. **(D)** Differences of five genes in GSE28042. **(E–I)** Survival analysis of *FOXO1, IRGM, MAPK1, MYC*, and *PRKCQ*. *p< 0.05, **p< 0.01, and ***p< 0.001.

We identified 11 genes associated with IPF prognosis from 71 autophagy-associated differential genes using univariate Cox analysis. These 11 genes can be considered prognostic marker genes for IPF (*BAG3, BCL2, BNIP3, EIF4EBP1, FOXO1, IRGM, ITGA6, MAPK1, MYC, PEX3, PRKCQ*) (**Figure 2A**). We used LASSO regression analysis to further reduce the number of genes in the signature. We constructed a 5-autophagy-associated genes signature based on the optimal value of λ (**Figures 2B, C** **)**.

The risk score was calculated as follows: risk score =*MAPK1*×1.651141668−*FOXO1*×0.162139386−*IRGM*×0.173049268−*MYC*×0.132639078−*PRKCQ*×0.133252635. Among the 5-autophagy-associated genes signature, *MAPK1* expression was higher in IPF patients than in controls, and *FOXO1*, *IRGM*, *MYC*, and *PRKCQ* expression was lower than in controls (**Figure 2D**). Survival analysis showed that the expression of *FOXO1*, *IRGM*, *MYC*, and *MAPK1* were able to predict the prognosis of IPF patients (**Figures 2E** **–H**). And the expression of *PRKCQ* was not able to predict the prognosis of IPF patients (**Figure 2I**).

**Figure 3:**
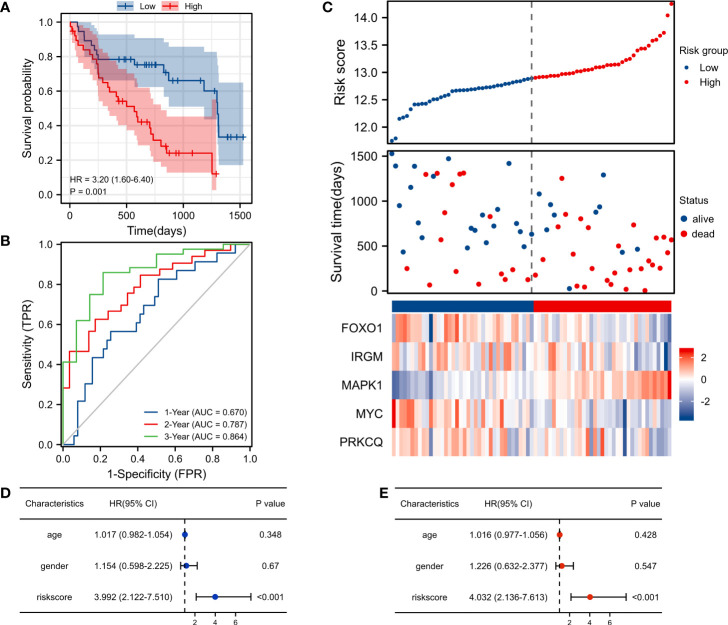
Risk score analysis of the autophagy-associated prognostic model in the GSE28042 training cohort. **(A)** Survival curve of high-risk score and low-risk score groups. **(B)** ROC curves evaluated the efficiency for predicting 1-, 2-, and 3-year survival. **(C)** Risk plot distribution, survival status, and heatmap of five genes expression. **(D)** Univariate cox analysis of riskscore in GSE28042. **(E)** Multivariate cox analysis of riskscore in GSE28042.

The K-M survival curve showed a significant difference in survival between the high-risk score group and the low-risk score group (p< 0.001), and the survival rate was lower in the high-risk group (**Figure 3A**). For 1-, 2-, and 3-year survival rates, the AUC predictive value of the AAG signature was 0.670, 0.787, and 0.864, respectively (**Figure 3B**). The risk map distribution and survival status of IPF patients showed that patients with high-risk scores had significantly lower survival times than patients with low-risk scores (**Figure 3C**). In addition, the heatmap of gene expression showed that *MAPK1* was highly expressed in the high-risk score group, while *FOXO1*, *IRGM*, *MYC*, and *PRKCQ* were more highly expressed in the low-risk score group (**Figure 3C**). Univariate and multivariate cox analysis have shown that riskscore can be used as an independent and valid indicator to assess the prognosis of IPF patients (**Figures 3D, E** **)**.

### Validation of the autophagy-associated prognostic model

**Figure 4:**
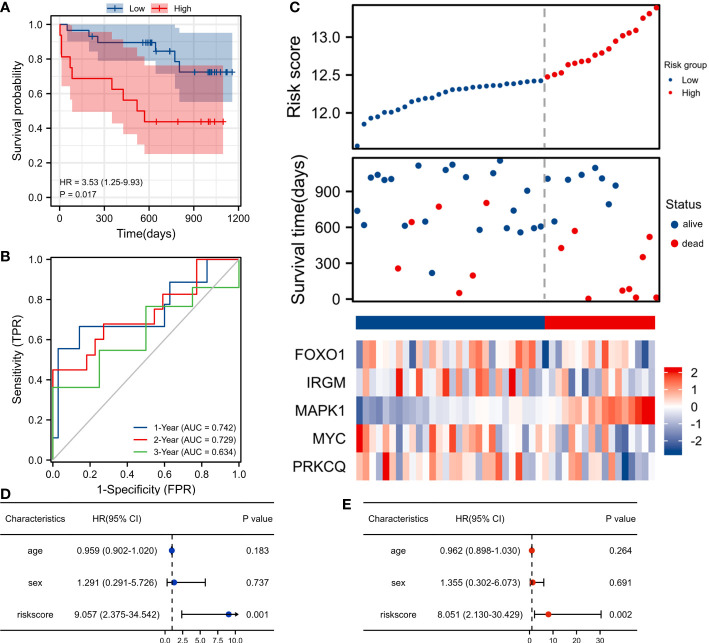
Validation of the autophagy-associated prognostic model in GSE27957. **(A)** Survival curve of high-risk score and low-risk score groups. **(B)** ROC curves evaluated the efficiency for predicting 1-, 2-, and 3-year survival. **(C)** Risk plot distribution, survival status, and heatmap of five genes expression. **(D)** Univariate cox analysis of riskscore in GSE27957. **(E)** Multivariate cox analysis of riskscore in GSE27957.

We included 45 patients with IPF from the GSE27957 database in the validation set cohort. In the validation cohort, Kaplan-Meier survival analysis showed that patients in the high-risk group had a shorter OS than those in the low-risk group(p<0.001) (**Figure 4A**). The AUC values for the 1-, 2-, and 3-year ROC curves in the validation cohort were 0.742, 0.729, and 0.634 respectively (**Figure 4B**). In the validation cohort, patients with IPF were also divided into the high-risk score group and the low-risk score group. Patients with high-risk scores had a worse survival status compared to patients with low-risk scores. In addition, we also plotted a heatmap showing the expression of the 5 AAGs (**Figure 4C**). In the validation dataset, univariate and multivariate cox analysis have shown that riskscore can be used as an independent and valid indicator (**Figures 4D, E** **)**.

### Construction of a nomogram based on five autophagy-associated genes

**Figure 5:**
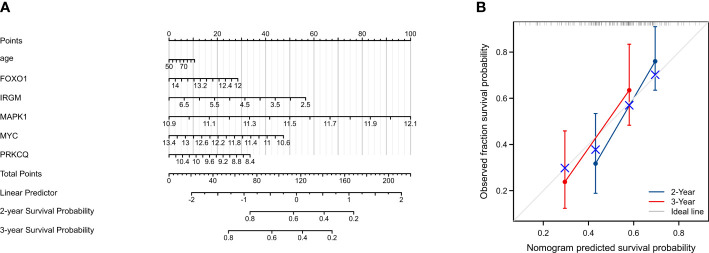
Construction and evaluation of a nomogram for predicting 2-, and 3-year overall survival rates of IPF patients. **(A)** Nomogram for predicting 2-, and 3-year overall survival of IPF patients. **(B)** Calibration curves showing the probability of 2-, and 3-year overall survival between the prediction and the observation.

To establish a quantitative approach to the prognosis of IPF patients, we constructed a predictive nomogram based on age and five screened AAGs (**Figure 5A**). The calibration curve shows that the predicted values and true values are in good agreement (**Figure 5B**). Therefore, the nomogram based on age and five AAGs presented a good accuracy for predicting 2- and 3-year survival of IPF patients.

### Relationship between autophagy-associated prognostic model and immune cell infiltration in IPF

**Figure 6:**
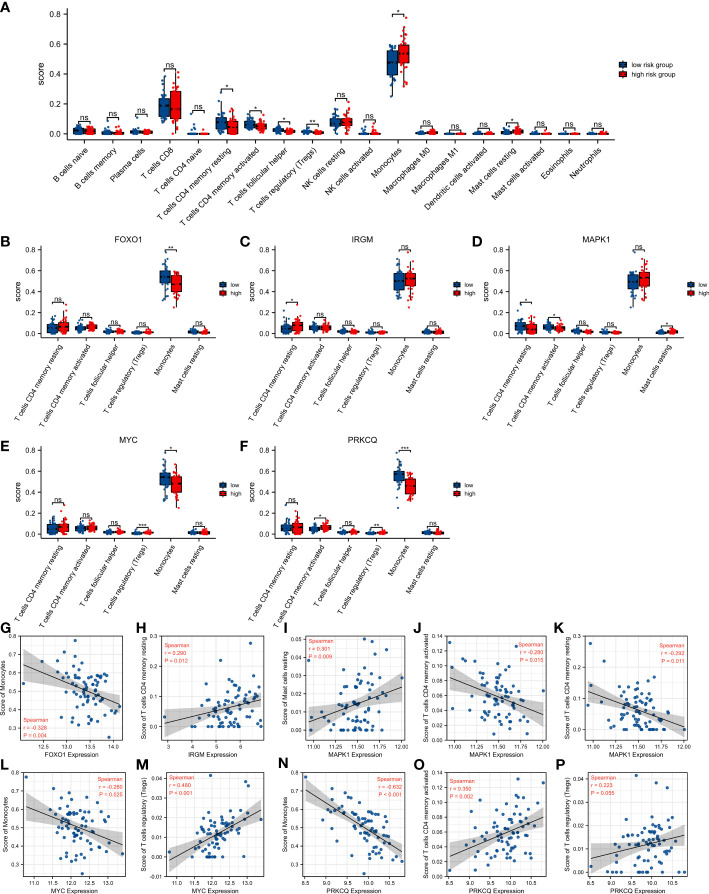
Relationship between autophagy-associated prognostic model and immune cell infiltration in IPF. **(A)** The difference of 22 immune cells between the high-risk score group and the low-risk score group. **(B–F)** Comparison of infiltration levels of 6 immune cells according to *FOXO1* **(B)**, *IRGM* **(C)**, *MAPK1* **(D)**, *MYC* **(E)**, and *PRKCQ* **(F)** expression levels. **(G–P)** The correlation analysis between autophagy-associated genes and specific immune cells. *p< 0.05, **p< 0.01, ***p< 0.001, and ns, no significance.

We calculated the difference in the level of infiltration of 22 immune cells between the high-risk score group and the low-risk score group using the CIBERSORT algorithm (**Figure 6A**). The IPF patients in the high-risk score group had lower T cells CD4 memory resting, T cells CD4 memory activated, T cells follicular helper, and T cells regulatory (Tregs), and had higher Monocytes and Mast cells resting than in the low-risk score group. Subsequently, we explored the relationship between the seven immune cells mentioned above and the five AAGs in the prognostic model. IPF patients with high *FOXO1* expression had lower levels of Monocytes (**Figure 6B**). And IPF patients with high *IRGM* expression had higher levels of T cells CD4 memory resting (**Figure 6C**). IPF patients with high *MAPK1* expression had lower levels of T cells CD4 memory resting and T cells CD4 memory activated, and higher Mast cells resting (**Figure 6D**). And IPF patients with high *MYC* expression had higher levels of T cells regulatory (Tregs) and lower Monocytes (**Figure 6E**). In addition, IPF patients with high *PRKCQ* expression had higher levels of T cells CD4 memory activated and T cells regulatory (Tregs), and lower Monocytes (**Figure 6F**). In addition, we provided the correlation analysis between autophagy-associated genes and specific immune cells. All correlations between five DEGs and specific immune cells were statistically significant, except for the correlation between *PRKCQ* and T cells regulatory (Tregs), which was not statistically significant (**Figures 6G** **–P**).

### Functional enrichment analysis of autophagy-associated prognostic model

**Figure 7:**
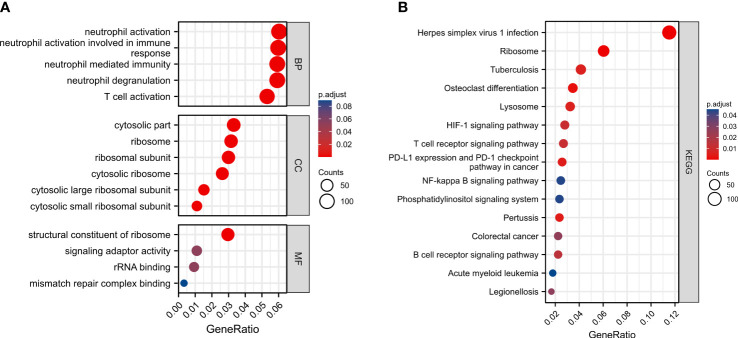
Functional enrichment analysis of autophagy-associated prognostic model between high-risk and low-risk score groups. **(A)** GO enrichment. **(B)** KEGG pathway analyses.

To reveal potential biological functions and pathways associated with the autophagy-associated prognostic model, we analyzed differential genes between high-risk and low-risk score groups using GO enrichment and the KEGG pathway analyses. GO enrichment revealed immune-related pathways, such as neutrophil activation, neutrophil mediated immunity, and T cell activation (**Figure 7A**). And KEGG pathway analyses were also enriched to inflammation-related pathways, such as T cell receptor signaling pathway, B cell receptor signaling pathway, and NF-kappa B signaling pathway (**Figure 7B**).

### Verification of the differentially expressed autophagy-associated genes in clinical samples and cellular models

**Figure 8:**
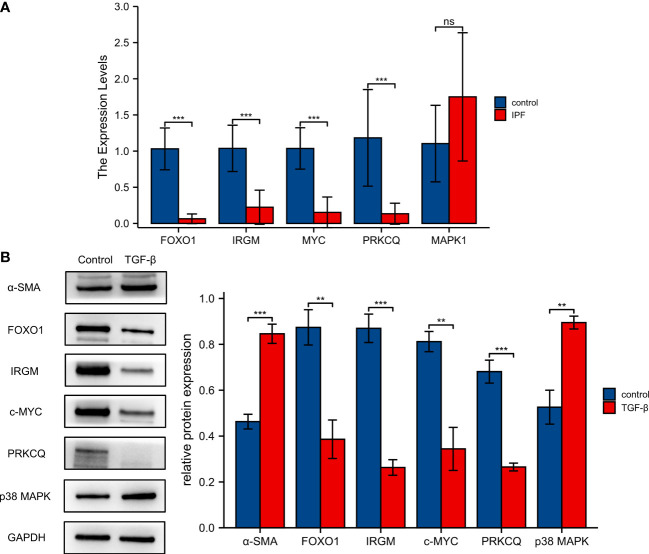
Validation of five screened autophagy-associated genes. **(A)**Validation of mRNA expression levels of *FOXO1*, *IRGM*, *MYC*, *PRKCQ*, and *MAPK1* with qRT-PCR. **(B)** The protein expression of *FOXO1, IRGM, MAPK1, MYC*, and *PRKCQ* was detected by western blotting. **p< 0.01, ***p< 0.001, and ns, no significance.

To further validate the reliability of the autophagy-associated prognostic model, we used clinical samples to further analyze the expression of five autophagy-associated genes. The results of qRT-PCR showed that the expression levels of *FOXO1*, *IRGM*, *MYC*, and *PRKCQ* were significantly decreased in IPF PBMC samples than in normal PBMC samples. However, *MAPK1* expression was not significantly different between the IPF and normal groups (**Figure 8A**). We used western blotting to assay the expression levels of 5 proteins in the autophagy-associated prognostic model. Elevated α-SMA expression demonstrates the success of the IPF cell model. The expression of FOXO1, IRGM, c-MYC, and PRKCQ was significantly lower in the TGF-β stimulated MRC-5 cell. And p38 MAPK was upregulated after stimulated by TGF-β (**Figure 8B**).

## Discussion

IPF is a highly heterogeneous disease, and the natural history of IPF patients is highly variable. Some patients with IPF deteriorate rapidly, while others have a slow progression (22, 23). It is difficult to predict the course and prognosis of individual patients (24). Over some time, several studies have been performed using clinical, imaging, and pathological indicators to predict progression and survival in IPF (25, 26). However, due to the retrospective nature of the studies and the uncertainty of the metrics, the accuracy of the predictors in these studies is various (24). Therefore, the development of appropriate and accurate prognostic models for IPF is urgent for clinical work.

In line with our study, the study by Huang et al. also constructed a prognostic model of autophagy-associated genes, which proved to be effective in predicting the prognosis of IPF patients (27). In our study, we used two GEO datasets to establish and validate our risk score prognostic model. And the samples were PMBC from IPF patients. In addition, we use the Human Autophagy Database website to determine the autophagy-associated genes. In our study, we first identified autophagy-associated genes associated with the prognosis of IPF patients. At first, we identified 11 autophagy-associated genes associated with IPF prognosis. Subsequently, we constructed an autophagy-associated prognostic model consisting of five genes using LASSO regression analysis. K-M survival curves and ROC curves demonstrated that the prediction model had good predictive power. Also, the K-M survival curves and ROC curves demonstrated that the model also showed excellent discrimination in the validation dataset. After that, we constructed a nomogram based on the expression of *FOXO1, MAPK1, IRGM, MYC*, and *PRKCQ* to predict the IPF patient prognosis directly.

*MYC*, also called *c-Myc*, is one of the major transcription factors involved in genomic regulation. *MYC* is involved in the regulation of fundamental biological processes such as cell proliferation, differentiation, metabolism, and apoptosis (28–30). *MYC* is involved in autophagosome formation in the early stages of autophagy, and *MYC* regulates autophagy through the JNK1-Bcl2 pathway and ROS. In contrast, *MYC* inhibition leads to defective autophagosome formation, which results in a decrease in autophagy (31). Aberrant activation of *MYC* in cancer has been extensively studied (32). In endothelial cells, downregulation of *MYC* activates a pro-inflammatory senescence phenotype and can act as a regulator of inflammation and endothelial dysfunction (33, 34). The expression of *MYC* in endothelial cells is protective in diet-induced liver inflammation and fibrosis (35). In a TGF-β-induced lung fibrosis model in rats, TGF-β inhibited cell proliferation by suppressing *MYC* expression (36). In addition, in a study on IPF patients, *MYC* was significantly lower in alveolar epithelial type II cells of IPF patients compared to controls (37). In our study, *MYC* expression was decreased in IPF patients and was seen as a protective factor in the risk model. Thus, the downregulation of *MYC* may play an important role in the development of IPF.

Mitogen-activated protein kinase 1(*MAPK1*), also known as extracellular signal-regulated kinases (ERKs), is an integration point for multiple signaling pathways (38). *MAPK1* is involved in a variety of biological processes that including cell proliferation, cell adhesion, cell migration, cell differentiation, metabolism, and transcription (39–41).MAP kinases are activated after phosphorylation of upstream kinases, which translocate to the nucleus after phosphorylation to activate a series of transcription factors, synthesize effector proteins and achieve corresponding changes in cellular state (40). In a study of emphysema, ERK activity was significantly increased in the lung tissue of patients compared to control subjects (42). And the activation of ERKs was closely associated with emphysema-related airway inflammation (43). In the BLM-induced lung fibrosis model, the phosphorylation level of ERK1/2 protein was elevated (44). And inhibition of ERK1/2 signaling attenuated BLM pulmonary fibrosis by inhibiting EMT (45). The increase of p-ERK1/2 and downstream-related targets was also observed in the lung tissue of IPF patients (46). In the autophagy-associated prognostic model, elevated *MAPK1* levels were associated with a worse prognosis in IPF. Thus, our study further confirms the role of *MAPK1* in promoting IPF progression.

*IRGM1* is a member of the interferon-inducible GTPases (IRGs) family, which is one of the strongest early resistance systems in cells (47). In inflammatory and infectious diseases, *IRGM1* is a core protein that promotes autophagic clearance of microorganisms and anti-inflammation (48, 49). In *IRGM1*-depleted cells, the cGAS-STING and RIG-I-MAVS signaling pathways were activated, which in turn increased the level of IFN responses (50). In LPS-stimulated murine macrophages, *IRGM1* was able to negatively regulate TLR4 signaling and prevent the overproduction of pro-inflammatory cytokines (51). In addition, *IRGM1-/-* mice exhibited lymphocytic infiltration in a variety of mucosal tissues, including lung tissue (52). In genome-wide association studies, mutations in *IRGM* were strongly associated with susceptibility to Crohn’s disease and tuberculosis (53, 54). Thus, decreased *IRGM1* may play an important role in IPF by promoting autophagy and inflammatory responses.

Protein kinase C theta (*PRKCQ*) is a serine/threonine kinase belonging to the PKC subfamily (55). Ca^2+^-dependent activation of *PRKCQ* is required in endoplasmic reticulum stress-induced autophagy (56). In the immune system, dysfunction of *PRKCQ* leads to the development of would autoimmune and inflammatory diseases (57). *PRKCQ* is highly expressed in T cells and is required for T cell-mediated responses (57, 58). *PRKCQ* expression is also decreased in lung adenocarcinoma and renal cell carcinoma compared to controls (59, 60). In the LPS-induced acute lung injury model in mice, inhibition of *PRKCQ* attenuated lung injury in mice by inhibiting Th17 cell responses through the Notch signaling pathway (61). And Th17 cells were reported to promote lung inflammation and fibrosis in a mouse lung fibrosis model (62, 63). However, studies of *PRKCQ* in IPF patients have not been reported. In our study, *PRKCQ* expression was found to be downregulated in IPF tissues and was associated with poor prognosis in IPF.

Forkhead transcription factors (*FOXOs*) family is considered to be key factors in cell fate regulation (64, 65). Activated *FOXOs* promote the expression of a variety of target genes that regulate various cellular processes including autophagy, cell cycle, and apoptosis (66, 67). Among them, *FOXO1/3* are the two most widely studied isoforms and play an important role in a variety of fibrotic diseases (68). Upregulation of *FOXO1* can attenuate renal tubular interstitial fibrosis and apoptosis in diabetic nephropathy by inhibiting STAT1 (69). In hepatic fibrosis, FOXO1 inhibits the activation, transdifferentiation, and proliferation of hepatic stellate cells (68). Ginsenoside attenuates hepatic fibrosis injury and inflammation by increasing the nuclear transport of *FOXO1 (* 70). In the fibroblasts of IPF patients, low levels of *FOXO3* favored the reduction of fibroblast autophagic activity and the maintenance of cell viability (71). However, the role of *FOXO1* in IPF has not been reported in studies. In autophagy-associated prognostic model, *FOXO1* is considered a protective factor in IPF. Decreased *FOXO1* may play an important role in the disease progression of IPF.

Differential gene function enrichment analysis between the high-risk and low-risk groups showed that neutrophil activation, neutrophil mediated immunity, and T cell activation are significantly activated in the high-risk group. Similarly, KEGG pathway analyses enriched immune-related pathways. And there was also a significant difference in immune cell infiltration between the high-risk and low-risk groups (T cells CD4 memory resting, T cells CD4 memory activated, T cells follicular helper, T cells regulatory, Monocytes and Mast cells resting). CD4 T cells may regulate pulmonary fibrosis in multiple ways (63, 72). Secretion of IFN-γ, IL-12, and TNF-α by Th1 cells was associated with attenuation of fibrosis (73–75). IL-4, IL-5, and IL-13 secreted by Th2 cells are also associated with the development of fibrosis (76, 77). Therefore, CD4 T cells can be anti-fibrotic or pro-fibrotic, depending on the state of the inflammatory environment (72). In the early stages of pulmonary fibrosis, T cells regulatory (Tregs) play a pro-fibrotic role by increasing TGF-β1 release and collagen deposition. While in the late stages of IPF, Tregs can inhibit the progression of pulmonary fibrosis (78). In the mouse model, Tregs reduced fibroplasia due to acute lung injury by decreasing fibroblast recruitment (79). Furthermore, an increase in the proportion of activated Tregs in the peripheral blood of IPF patients was negatively correlated with the severity of the disease (80). Monocytes play a key role in fibrogenesis through the release of pro-fibrotic inflammatory cytokines (63). Increased numbers of monocytes have also been shown to be associated with poor prognosis in IPF in several studies (81, 82). In addition, monocytes are progenitors of pro-fibrotic macrophages and fibroblasts (83, 84). Mast cells have a large amount of pro-fibrotic cytokines in their granules (85, 86). The number of mast cells was increased in the lung tissue of IPF patients, and the products of mast cells were increased in BALF (87, 88). In addition, mast cells may promote the fibrotic process by stimulating fibroblasts in the lung (89). Therefore, we hypothesize that genes in autophagy-associated prognostic model may be involved in the development of IPF by regulating these immune cells.

In conclusion, our study constructed and validated an autophagy-associated prognostic model based on *MYC, MAPK1, IRGM, PRKCQ*, and *FOXO1*. We also found that *MYC, MAPK1, IRGM, PRKCQ*, and *FOXO1* may influence the progression of IPF by regulating immune responses and immune cells. In addition, the results of qRT-PCR showed that the expression of *FOXO1*, *IRGM*, *MYC*, and *PRKCQ* was decreased in PBMC of IPF patients compared to normal samples. The expression of FOXO1, IRGM, c-MYC, and PRKCQ was significantly lower and p38 MAPK was upregulated in the TGF-β stimulated MRC-5 cell. However, our study also has some limitations. First, we need to follow up in a larger sample size population to validate the accuracy of the autophagy-associated prognostic model. The small sample size may make the difference of *MAPK1* between the IPF patient and control groups insignificant. Our study also did not include drug use in IPF patients. As a recommended drug for the treatment of IPF, the use of pirfenidone may inhibit *MAPK1* gene expression (90). In addition, we need further functional and mechanistic experiments to explore the potential mechanisms underlying the role of autophagy-associated genes in IPF progression.

## Data availability statement

The datasets presented in this study can be found in online repositories. The names of the repository/repositories and accession number(s) can be found below: https://www.ncbi.nlm.nih.gov/geo/, GSE28042 https://www.ncbi.nlm.nih.gov/geo/, GSE27957.

## Ethics statement

The studies involving human participants were reviewed and approved by The second hospital of Shandong University Ethics Committee. The patients/participants provided their written informed consent to participate in this study.

## Author contributions

ZY, FGQ and LJJ designed the entire study. FGQ, and LJJ completed the experiments. ZY and FGQ performed the data analysis and wrote the article. FGQ, LJJ, WZ and LCY collected the data and revised the manuscript. FGQ and LJJ contributed equally to this work and should be considered the first authors. All authors approved the final manuscript. All authors contributed to the article and approved the submitted version.
